# Prediction of Left Ventricular Mechanics Using Machine Learning

**DOI:** 10.3389/fphy.2019.00117

**Published:** 2019-09-06

**Authors:** Yaghoub Dabiri, Alex Van der Velden, Kevin L. Sack, Jenny S. Choy, Ghassan S. Kassab, Julius M. Guccione

**Affiliations:** 1 California Medical Innovations Institute, San Diego, CA, United States,; 2 Department of Surgery, University of California, San Francisco, San Francisco, CA, United States,; 3 SIMULIA, Dassault Systemes, Johnston, IA, United States,; 4 Division of Biomedical Engineering, Department of Human Biology, University of Cape Town, Cape Town, South Africa

**Keywords:** left ventricle, machine learning, finite element method, XGBoost, Cubist

## Abstract

The goal of this paper was to provide a real-time left ventricular (LV) mechanics simulator using machine learning (ML). Finite element (FE) simulations were conducted for the LV with different material properties to obtain a training set. A hyperelastic fiber-reinforced material model was used to describe the passive behavior of the myocardium during diastole. The active behavior of the heart resulting from myofiber contractions was added to the passive tissue during systole. The active and passive properties govern the LV constitutive equation. These mechanical properties were altered using optimal Latin hypercube design of experiments to obtain training FE models with varied active properties (volume and pressure predictions) and varied passive properties (stress predictions). For prediction of LV pressures, we used eXtreme Gradient Boosting (XGboost) and Cubist, and XGBoost was used for predictions of LV pressures, volumes as well as LV stresses. The LV pressure and volume results obtained from ML were similar to FE computations. The ML results could capture the shape of LV pressure as well as LV pressure-volume loops. The results predicted by Cubist were smoother than those from XGBoost. The mean absolute errors were as follows: XGBoost volume: 1.734 ± 0.584 ml, XGBoost pressure: 1.544 ± 0.298 mmHg, Cubist volume: 1.495 ± 0.260 ml, Cubist pressure: 1.623 ± 0.191 mmHg, myofiber stress: 0.334 ± 0.228 kPa, cross myofiber stress: 0.075 ± 0.024 kPa, and shear stress: 0.050 ± 0.032 kPa. The simulation results show ML can predict LV mechanics much faster than the FE method. The ML model can be used as a tool to predict LV behavior. Training of our ML model based on a large group of subjects can improve its predictability for real world applications.

## INTRODUCTION

According to the American Heart Association 2019 Update [1], the prevalence of heart failure (HF) has increased from 5.7 million (2009 to 2012) to 6.2 million (2013 to 2016) in Americans older than 20 years of age. This prevalence is projected to increase 46% by 2030 [2]. In 2012, the total cost for HF was ~$31 billion and it is estimated that by 2030, the total cost will increase to $70 billion [2]. Therefore, there is substantial need for innovative treatment strategies for HF. Computational simulation provides a virtual platform where the behavior of the heart can be simulated and novel interventions can be assessed. Such simulations provide key insights on how HF develops, and how pharmaceutical and device design and implantation can be optimized. Among computational simulation, the finite element (FE) method has been extensively used by our group [3–5]. One important example of using FE to characterize HF is to understand the etiology of Heart Failure with Preserved Ejection Fraction (HFpEF) [4, 6]. We have used four-chamber FE models for the mechanical analysis of the heart [7, 8].

One shortcoming of the FE model is that it typically requires a relatively long time to run. For example, the four-chamber heart model developed in 2015 [7, 9] required 1,000 CPU hours to converge for a 1 s cardiac cycle. Today (2019), this time has been reduced to 100 CPU hours, but even with these improved models, the estimated time to tune a model would still be thousands of CPU hours. This provides an incentive to study the feasibility of a reduced order model based on machine learning (ML). In addition, ML models can be used to estimate initial conditions to speed up FE model convergence.

The health conditions of the left ventricle (LV) are strongly linked to stresses [10]. Myocardial stresses determine the metabolic requirements of the heart and are important stimuli for growth and remodeling of the myocardium. Furthermore, the blood flow in the coronary arteries is influenced by the stresses in the surrounding cardiac tissue. Therefore, determination of LV stress is informative for better understanding the diseased conditions or the recovery status of the myocardium after treatment. We have used FE models to calculate stresses in the LV [4, 8], but the time required is relatively too long for real-time applications. This shortcoming of LV FE modeling prevents clinical implementation.

In the machine design industry, computational time for numerical analysis cannot be too long to be used during design iterations. To overcome this problem, ML has been used for model-based system design, for example, in the automobile industry [11]. Recently, ML models have been reported for vascular applications [12–14]. Additionally, cardiac mechanics have been studied using ML [15, 16]. Using ML models, the behavior of the LV in response to alterations in material properties, loads, boundary conditions etc. can be predicted in nearly real time. This relatively fast prediction of the LV behavior could provide a tool for real-time monitoring of the LV behavior with applications in cardiac devices design, monitoring the health conditions of the LV, etc.

Therefore, the goal of this paper was to develop an FE-based ML platform to simulate LV mechanics. To the best of our knowledge, the decision tree algorithm, eXtreme Gradient Boosting (XGboost) [17–19] has not been used for cardiac mechanics. This ML algorithm has been recognized in terms of accuracy, flexibility and speed [19, 20]. We also used Cubist, a similar package with smoother predictions, for LV pressure and volume. The resultant ML model can provide myocardial stresses in seconds and can be used in iterative medical device design for the heart. Abaqus FE software was used to generate 77 LV models for pressure and volume and 100 models for stress prediction training. The features of the ML model were mechanical properties and time for pressure and volume predictions, and mechanical properties and elements centroid coordinates for stress predictions. The FE models were used to create ML models that reduced the LV pressure and volume prediction time from nearly 1,000 CPU hours to 11 CPU seconds, and stress prediction time from nearly 20 CPU minutes to 5 CPU seconds.

## METHODS

### Finite Element Models

*In vivo* data were obtained under a protocol approved by our institutional review board [21]. The geometries, material behavior, loads and boundary conditions (BCs) were implemented as follows. We used two geometries. For pressure and volume predictions, we used data from a four-chamber model including LV, right ventricle (RV), left atrium, and right atrium. For stress predictions we used data from a swine LV-only model. The specifications of geometry reconstructions have been previously reported [9, 21]. These FE models consider LV as a passive material during diastole, and as a contractile material during systole. The constitutive equation of the passive and active behavior has been extensively described [4, 8, 9]. Briefly, the passive behavior described the tissue as a hyperelastic fiber-reinforced material, as follows:

(1) $$\Psi_{dev}=\frac{a}{2b}e^{b\left(I_{1}-3\right)}+\sum_{i=f,s}\frac{a_{i}}{2b_{i}}\left\{e^{b_{i}{\left(I_{4i}-1\right)}^{2}}-1\right\}+\frac{a_{fs}}{2b_{fs}}\left\{e^{b_{fs}{\left(I_{8fs}\right)}^{2}}-1\right\}\;\Psi_{vol}=\frac{1}{D}\left(\frac{J^{2}-1}{2}-\text{ln }(J)\right)$$

where *a* and *b* are isotropic stiffness of the tissue, *a*_*f*_ and *b*_*f*_ are tissue stiffness in the fiber direction, and *a*_*fs*_ and *b*_*fs*_ are the stiffness due to the connection between fibers and sheet; *I*_*1*_, *I*_4*i*_ and *I*_8*fs*_ are invariants, defined as follows:

(2) $$I_{1}:=tr(C)\;I_{4i}:=C:\left(i_{0}⊗i_{0}\right)\;I_{8fs}:=C:sym\left(f_{0}⊗s_{0}\right)$$

where **C** is the right Cauchy-Green tensor, and ***f***_0_ and ***s***_0_ are vectors that define the fiber and sheet directions, respectively. *J* is the deformation gradient invariant, and *D* is a multiple of the Bulk Modulus *K* (i.e., $D=\frac{2}{K}$).

The active tissue behavior is described as follows [4, 8, 22, 23]:

(3) $$T_{0}=T_{\text{max}}\frac{Ca_{0}^{2}}{Ca_{0}^{2}+ECa_{50}^{2}}C_{t}$$

where *T*_max_ is the isometric tension at the largest sarcomere length and highest calcium concentration, *Ca*_0_ is the peak intracellular calcium concentration, and

(4) $$C_{t}=\frac{1}{2}(1-\text{cos}\omega ),$$

$$\omega =\{\begin{array}{c} \pi \frac{t}{t_{0}}\text{ when }0\leq t\leq t_{0}\\ \pi \frac{t-t_{0}+t_{r}}{t_{r}}\text{ when }t_{0}\leq t\leq t_{0}+t_{r}\\ 0\text{ when }t\geq t_{0}+t_{r} \end{array}$$

(5) $$t_{r}=ml+b$$

*m*, *b* are constants that specify the shape of the linear relaxation duration and sarcomere length relaxation, and *t*_0_ is time to reach peak tension after the initiation of active tension.

In addition,

(6) $$ECa_{50}=\frac{{\left(Ca_{0}\right)}_{\text{max}}}{\sqrt{\text{exp }\left[B\left(l-l_{0}\right)\right]-1}},l=l_{R}\sqrt{2E_{ff}+1}$$

where:

*E*_*ff*_= Lagrangian strain in the fiber direction,

*B* is a constant that specifies the shape of the peak isometric tension-sarcomere length relation,

*l*_0_ is the sarcomere length that does not produce active stress,

*l*_*R*_ is the sarcomere length with the stress-free condition, and (*Ca*_0_)_max_ is the maximum peak intracellular calcium concentration.

The passive stress is derived from passive behavior, during diastole, and the total stress is the sum of passive and active stresses during systole. We assumed homogeneous contraction (T_max_) in all models.

The specifications of the models, including loads and boundary conditions, have been described in our previous publications in detail [4, 7, 8]. Briefly, in LV models, the LV pressure was applied to the endocardial surface in early diastole. The interaction between LV and the arterial system was model using lumped parameter 1-D circulatory models. In this paper, the four-chamber heart FE results were obtained after several initial cycles, and FE data for LV pertain only to the diastole part of the cardiac cycle.

## ML Models

### The ML Model for LV Pressure and Volume Prediction

**FIGURE 1 |:**
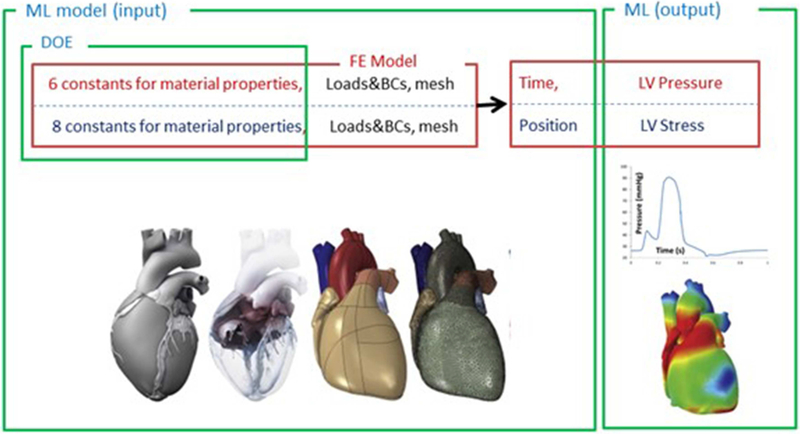
The structure of the ML model used to predict LV pressure, volume and stress. The loads, boundary conditions (BC), the mesh and material properties were parts of the FE model. The material properties (6 active properties for pressure and volume, 8 passive properties for stress predictions) were sampled by design of experiments (DOE) and used as the inputs of the ML model. For LV pressure and volume predictions, time (t) was another input of the ML model. For LV stress predictions, position of each element was also an input (stresses at end diastole were used).

The structure of the ML-FE surrogate model is shown in Figure 1. This was a supervised learning regression problem. We used a tree ensemble learning approach whereby XGboost package [17–19] in Python programming language was used to predict LV pressures and volumes based on material properties and time. The features of the ML model are listed in Table 3. We also used Cubist [24], a package that makes predictions using rules which are learned during training from decision trees. The Latin hypercube design of experiments (DOE) method was used to sample the features. Using the radial basis function (RBF) and lumped parameter simulations, the DOE was optimized to minimize the error due to sampling, and also minimize the number of FE simulations [25, 26]. The number of selected FE models for training and test data were 77 and 3, respectively. The test data were not included in the training data. It should be noted that each single training or test data for LV volume and pressure ML models, refers to a single time point in a cardiac cycle. Because each cardiac cycle was composed of 401 time points, each FE model included 401 data examples. The data for each FE model at different time points were not independent, but they were different features within one single cardiac cycle dataset [27]. The limits of the features were set as follows: 0.0015 < *l*_0_ < 0.0028, 0.075 < *t*_0_ <0.25, 0.65 < *T*_max_ <1.9. Only active properties were altered in the datasets used to predict LV pressure and volume. A hyperparameter grid search was conducted to find optimized parameters as indicated in section “ML Parameter Tuning and Error Estimation”.

### The ML Model for LV Stress

The passive material properties (*a, a*_*f*_ *, a*_*s*_*, a*_*fs*_) were altered using Latin hypercube DOE to produce 120 LV models. The range of these variables was as follows: 0.229e-3 < *a* < 9.881e-3, 0.005e-1 < *a*_*f*_ < 49.901e-3, 9.1e-05 < *a*_*s*_ < 6.986e-3, 7.568e-5 < *a*_*fs*_ < 3.952e-3 MPa. The ratios between passive properties *b, b*_*f*_ *, b*_*s*_*, b*_*fs*_ and corresponding *a, a*_*f*_ *, a*_*s*_ and *a*_*fs*_ were based on data in the literature [23]; consequently, values of *b, b*_*f*_ *, b*_*s*_*, b*_*fs*_ were obtained from corresponding *a, a*_*f*_ *, a*_*s*_*, a*_*fs*_. For these simulations, D (Equation 1) was 0.2 MPa^−1^ and the end diastolic pressure was 16.38 mmHg. There were 576 elements in the endocardium where element centroid stresses in fiber direction (S_11_), cross-fiber direction (S_22_), and shear stress (S_12_) were calculated using FE method. XGboost was used to predict stresses: 100 out of 120 FE models for training and 20 out of 120 FE models for testing (we did not use Cubist for stress predictions). It should be noted that for LV stress predictions, each single training or test example refers to a single finite element. Because there were 576 elements in each FE model, there were 576 training or test examples in each FE model. The data for each FE model at different elements were not independent, but they were different features within one single LV dataset [27]. Stresses at end diastole (ED) were used to implement ML predictions. The features of the ML model are summarized in Table 3.

### ML Parameter Tuning and Error Estimation

To select the optimized hyperparameters for ML estimators, a grid search analysis was conducted in XGBoost for the blood pressure and volume as well as stress predictions (Table 1). We used a decision tree algorithm for the base learner (booster = “gbtree”), and the learning task and the related objective were set by a linear regression analysis (objective = “reg:linear”). The score used to select the optimized parameters was the coefficient of determination, *R*^2^, calculated as follows:

(7) $$R^{2}(y,\hat{y})=1-\frac{\sum_{i=1}^{n}{\left(y_{i}-{\hat{y}}_{i}\right)}^{2}}{\sum_{i=1}^{n}{\left(y_{i}-\bar{y}\right)}^{2}}$$

where *y* and $\hat{y}$ are actual and predicted values, *n* is the number of data points, and

$$\bar{y}=\frac{1}{n}\sum_{i=1}^{n}y_{i}$$

The higher the *R*^2^ is, the better the predictability of the model is (Scikit-learnv.0.21.2 documentation). After the best model was found by the grid search, that model was used for predictions.

In addition to *R*^2^ we computed mean absolute error (MAE) for the results (Scikit-learnv.0.21.2 documentation):

(8) $$MAE=\frac{\sum_{i=1}^{n}\left|y_{i}-{\hat{y}}_{i}\right|}{n}$$

### Feature Importance

For the ML models, the relative importance of the features was calculated using the XGBoost package in Python (we used “Gain” for this purpose). The relative importance values are based on how often each feature was used for splitting weighted by the squared improvements in the model due to those splits, averaged over all trees [17, 28].

## RESULTS

**FIGURE 2 |:**
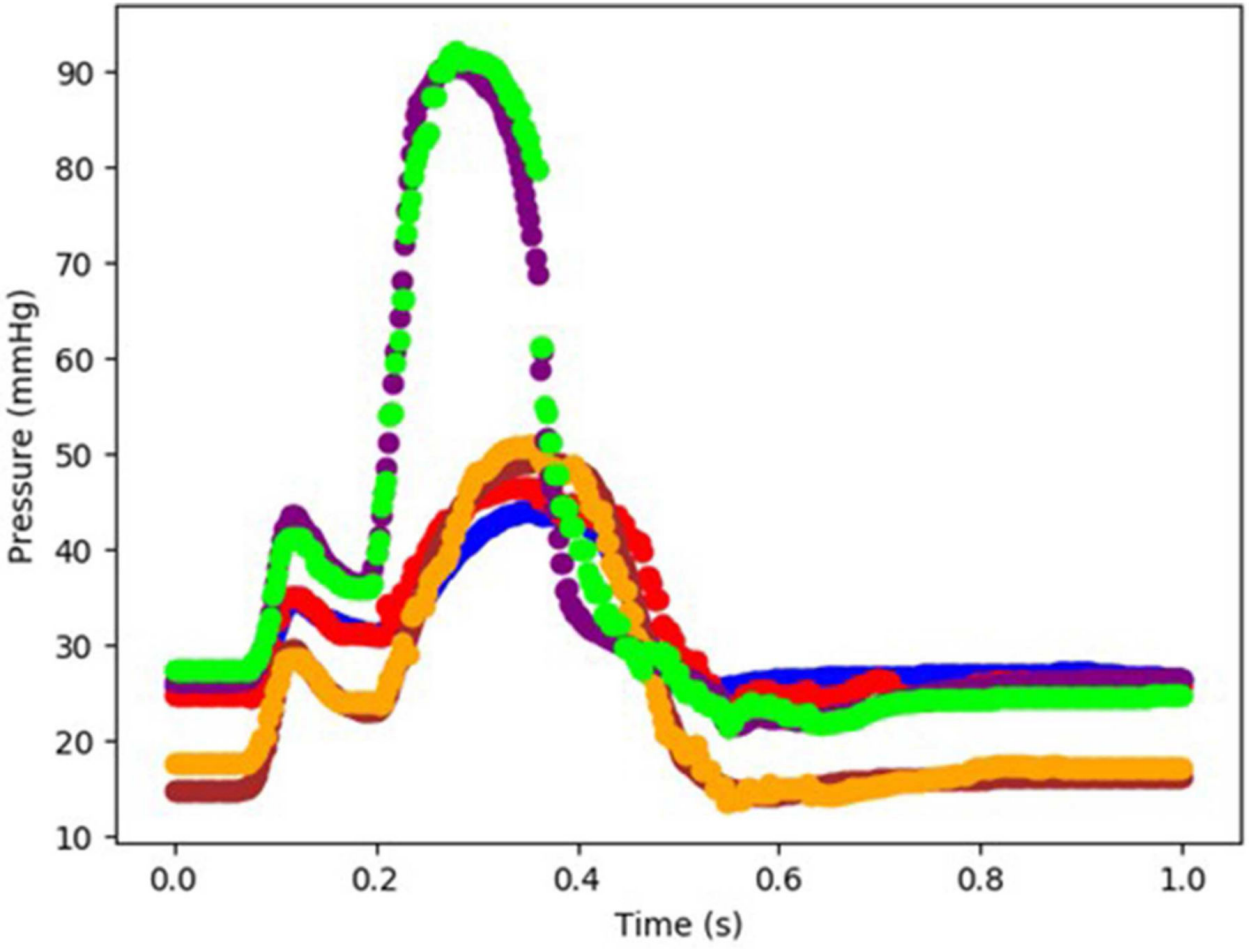
The FE-computed and ML-predicted LV pressure curve for three cycles (3 × 401 = 1,203 test data) based on random selection of mechanical properties (FE: blue, brown, purple, ML: red, orange, green, respectively). For these results, XGboost was used. The variability in three test cardiac cycles (1,203 test data each) can be seen in this figure.

**FIGURE 3 |:**
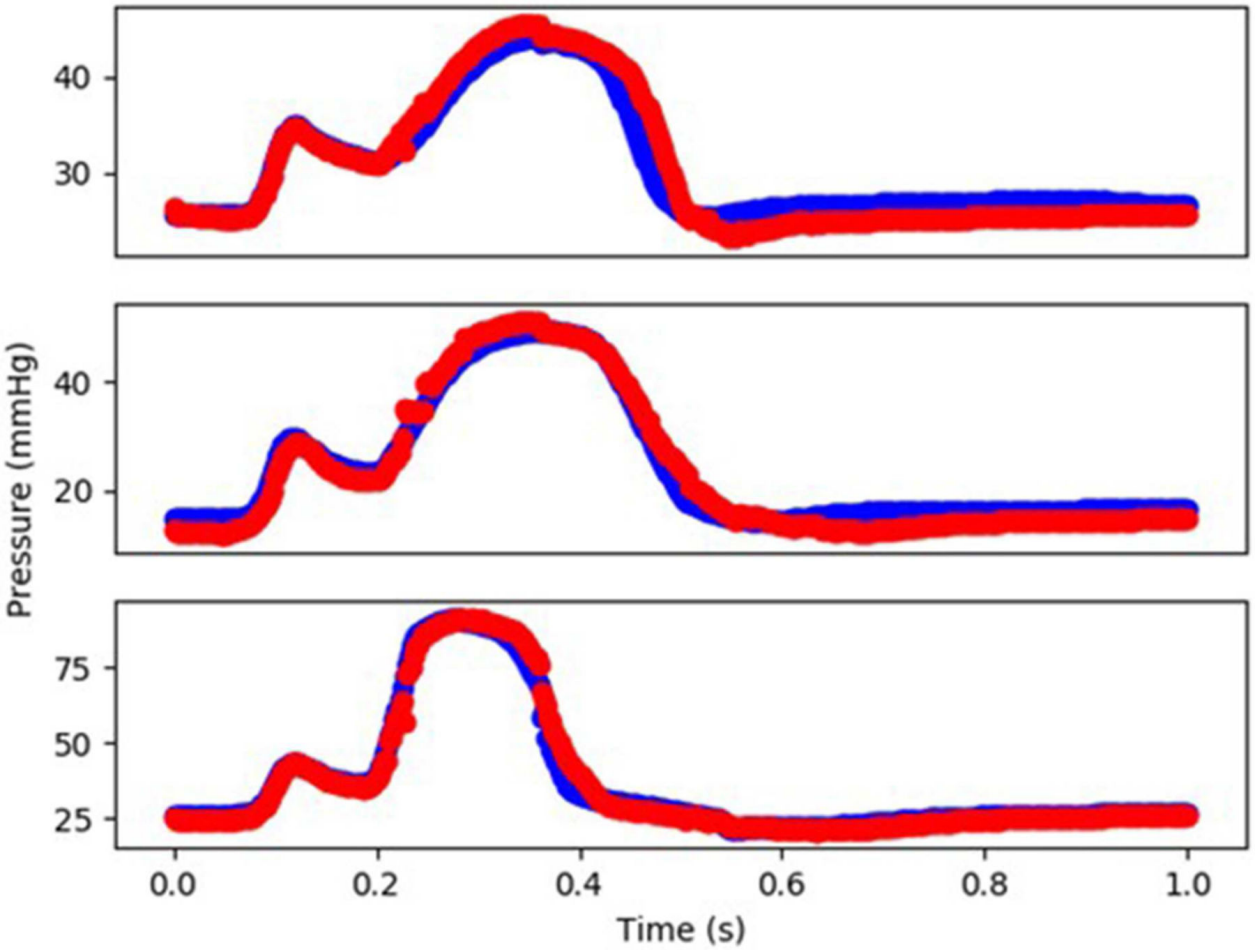
The FE-computed and ML-predicted LV pressure curve for three cycles (3 × 401 = 1,203 test data) based on random selection of mechanical properties (FE: blue, ML: red). For these results, Cubist was used.

**FIGURE 4 |:**
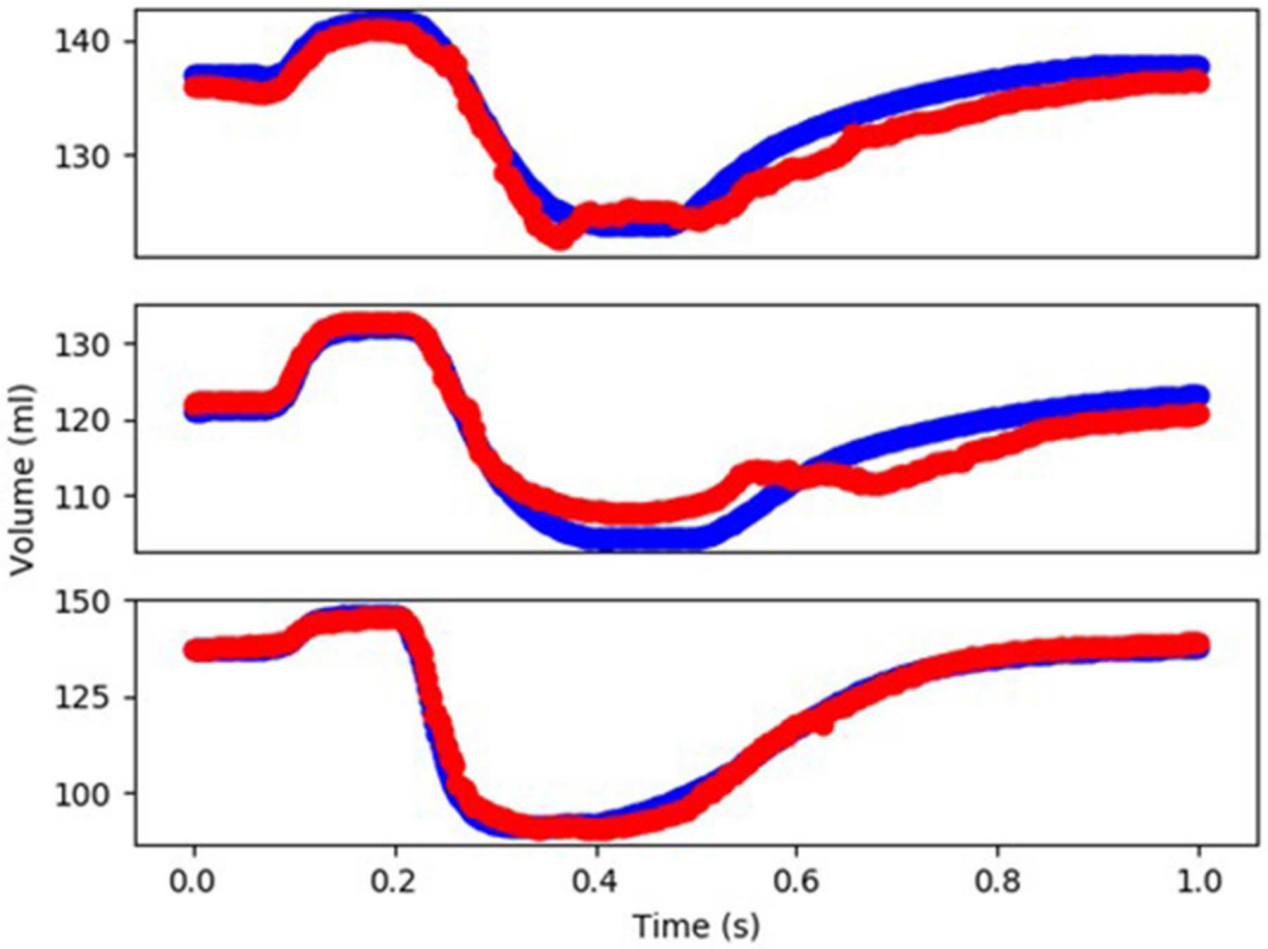
The FE-computed and ML-predicted LV volume curve for three cycles (3 × 401 = 1,203 test data) based on random selection of mechanical properties (FE: blue, ML: red). For this results XGBoost was used.

**FIGURE 5 |:**
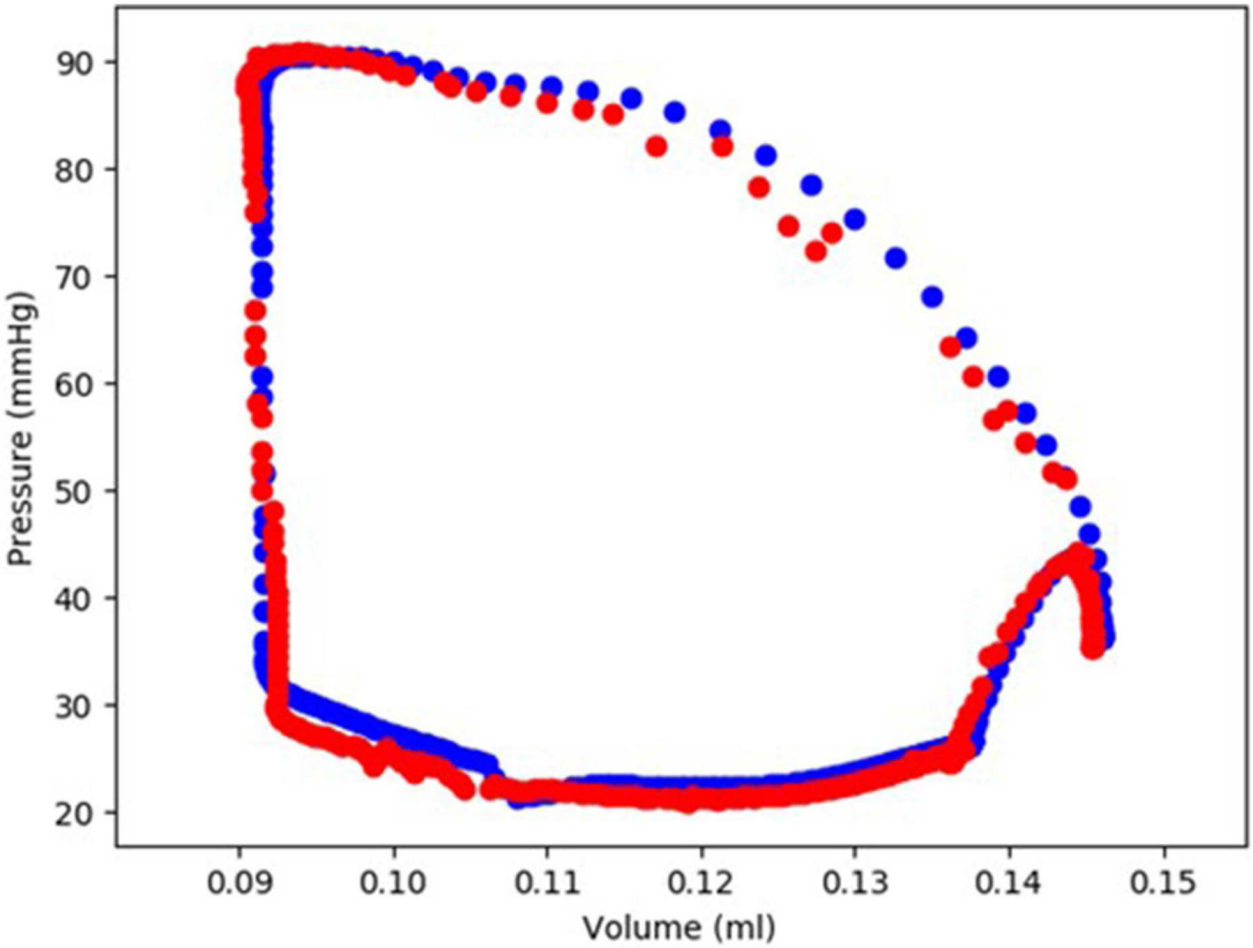
The FE-calculated and ML-predicted LV pressure-volume loop for one cardiac cycle (401 test data) based on random selection of active mechanical properties (FE: blue, ML: red). For this result, Cubist was used.

The XGBoost and cubist algorithms produced LV pressures in agreement with the FE computations in a much shorter time (Figures 2, 3, Tables 2, 5). The *R*^2^ scores (Equation 7) were relatively close to 1 for both Cubist and XGBoost (Table 5). When predicted pressures and volumes were used, the resultant pressure volume loop predicted by ML was in agreement with FE calculations (Figure 5). The results for volume predictions were also noticeably close to FE results (Figures 4, 5, Tables 4, 5). It should be noted that all results pertain to test data.

**FIGURE 6 |:**
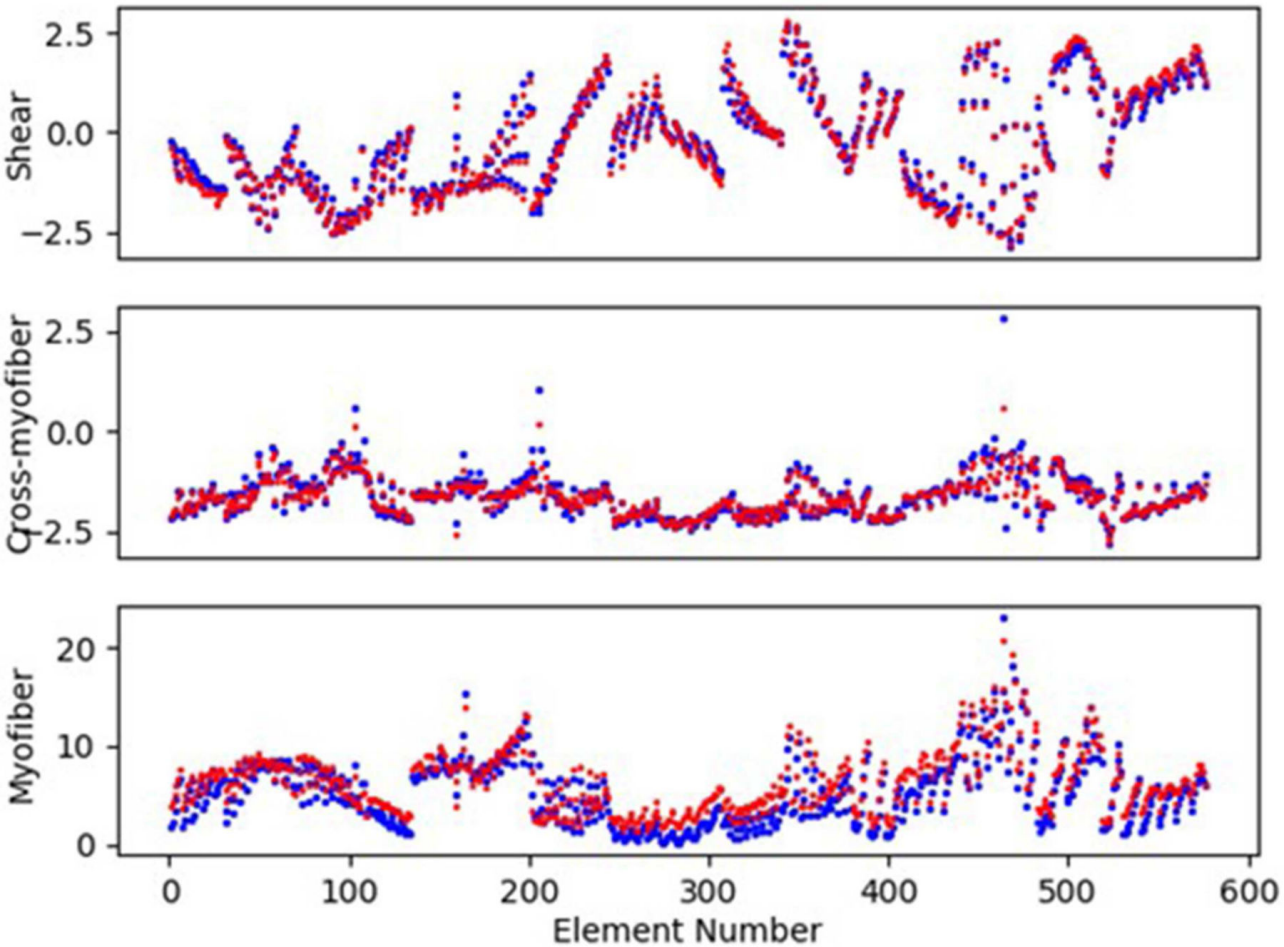
The LV endocardium stresses (kPa) computed by FE and predicted by ML for a sample test model (FE: blue, ML: red). The stresses pertain to the centroid of elements. For these results, XGboost was used.

**FIGURE 7 |:**
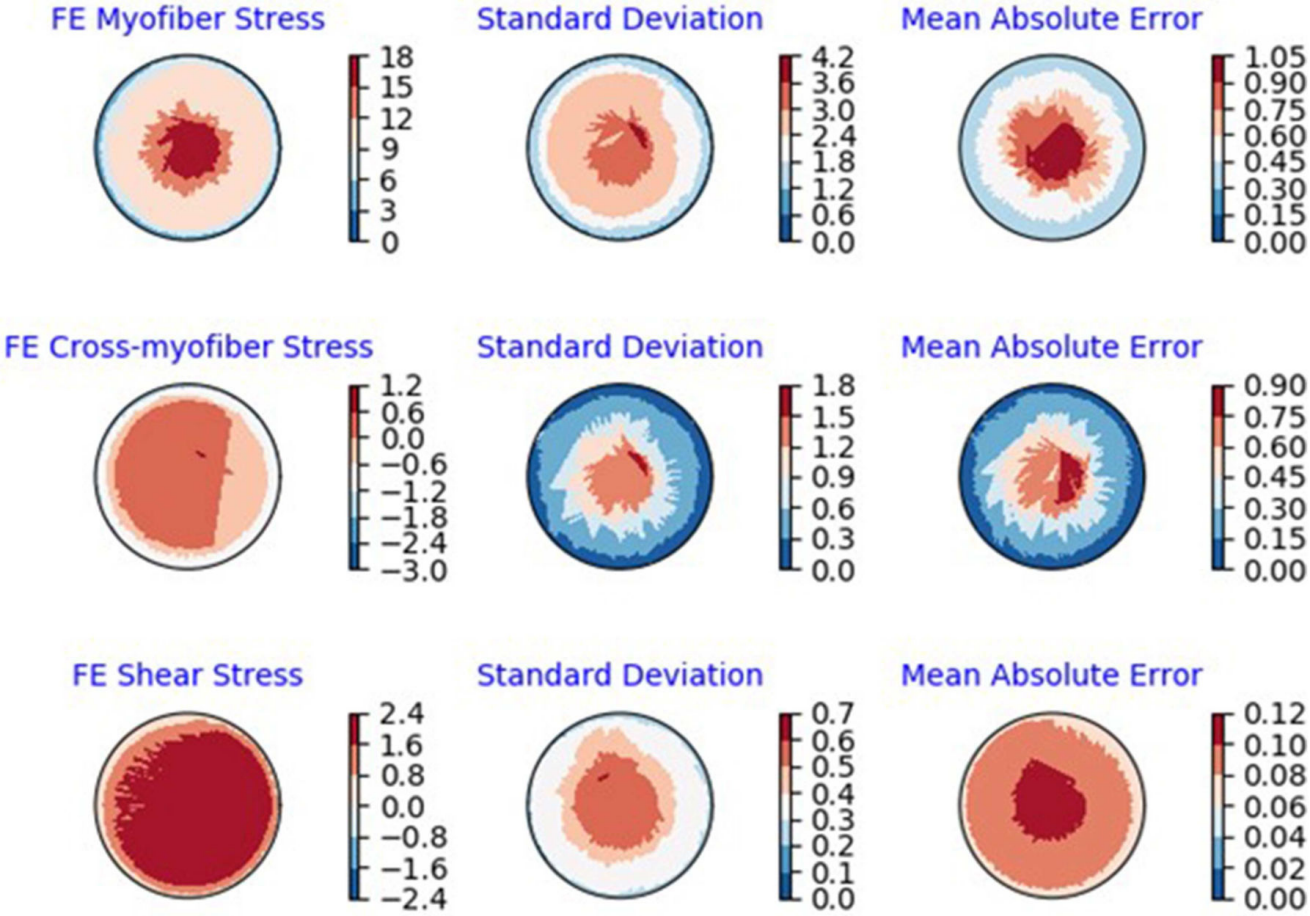
Polar plots of average: FE results, standard deviation of the FE results and MAE (kPa) between ML and FE results. The averages were calculated over all 20 test models.

The minimum and maximum and time of maximum pressure and volume were similar for FE and ML results, but the differences between maximum dP/dt and dV/dt for FE and ML models were noticeable (Table 4). Other aspects of LV pressure were also captured in the ML predictions, in particular, the bump before the contraction (Figure 2). The stresses predicted by ML were in agreement with FE calculations (Figures 6, 7). The regional variation of stresses can be predicted by ML with noticeable accuracy (Figure 7).

Results showed that for the features considered in LV pressure predictions, *l*_0_ in the RV plays the most important role, followed by time, *l*_0_ in the LV, and *t*_0_ in the LV. The other parameters that noticeably influence LV pressure in systole are *T*_max_ in the LV,*T*_max_ in the RV, and *t*_0_ in the RV and (Table 3). For LV volume predictions, *l*_0_ in the LV was the most important feature followed by time, *l*_0_ in the RV, *T*_max_ in the LV, *t*_0_ in the LV, *t*_0_ in the RV,*T*_max_ in the RV (Table 3).

For the myocardial stresses, the most important factor is the location where stress is computed. The passive parameters have different influences on myofiber (S_11_), cross-myofiber (S_22_), and shear stresses (S_12_). For S_11_, passive properties in the sheet direction are more important than passive properties in the fiber direction, whereas for S_22_, passive properties in the fiber direction are more important than those in the sheet directions. For shear stress, the passive properties in the sheet direction are more important than those in the fiber direction (Table 3).

## DISCUSSION

We used ML to predict LV pressures and volumes on the order of seconds. It took ~1000 CPU hours to compute LV pressures, but <12 CPU seconds to obtain the same data using the ML approach (Table 2). The results of the ML model agreed closely with those of the FE models (Tables 4, 5). Although ML has been used to analyse the mechanics of cardiac tissue [15, 16], to the best of our knowledge, ours is the first study that used decision tree algorithms, XGBoost and Cubist to compute LV pressure and volumes, as well as stresses. Using decision trees, XGBoost provides the importance of features in the predictions, which can be used to assess the role of features in the heart behavior.

We used a grid search analysis to determine optimized hyper-parameters for ML models (Table 1). We could use more parameters in grid search analysis, but the training time would increase. Because the FE and ML results are in reasonable agreement, using more hyper-parameters in the grid search would be inefficient computationally. Similarly, we could generate more FE data to feed to the ML model, but it would not be computationally efficient, because generating FE data is time-consuming. In other words, over-training the ML model by using more hyper-parameters in the grid search or generating more FE results was not computationally efficient.

Historically, the LV pressure has been modeled using different approaches, including lumped electrical circuit analogies [29] and FE [4, 8, 9]. Lumped electrical circuit analogies methods provide informative insights about the cardiovascular system, but cannot provide important information such as stresses in the LV. On the other hand, in FE models, the simulation time is usually too long to provide real-time information (Table 2). The ML approach presented here provides a fast and reliable (in comparison to FE models) modeling approach in real-time.

Comparison between actual and predicted LV pressure and volume parameters revealed quite reasonable agreement between the two (Table 4). The differences between actual and predicted dP/dt and dV/dt are due to the fact that we did not train the model for dP/dt and dV/dt. Moreover, we did not remove noise from ML predictions, which could lead to larger time derivatives (noise can be removed using a filter).

In our study, values of *R*^2^ were relatively close to 1 for LV pressure and volume predictions, indicating close agreement between our FE and ML results. However, the *R*^2^ function is not as informative as the FE and ML results in a time domain (Figures 2–4). For stress predictions, the values of *R*^2^ were also relatively high, which again indicates the ML and FE results are relatively in close agreement. A more informative comparison between FE and ML stress predictions can be done using Figure 7, which compares the FE and ML results with spatial distributions considered. The MAE values show relatively close agreement between FE and ML results (Table 5).

Although the XGBoost package provided relatively correct predictions, it produced jagged approximations for LV pressures (Figure 2). Since XGBoost uses a sequence of weak decision trees, it predicts the outputs in a discrete way [20]. Results from Cubist resolved this limitation as the volumes and pressures approximated by Cubist were noticeably smoother than those from XGBoost (Figure 3 vs. Figure 2). However, it would be trivial to add a filter to the XGB results in the time domain and make it similarly smooth. In fact XGBoost has several advantages compared to other gradient boosting algorithms, in terms of speed, flexibility for handling data, sensitivity to outliers, and performance [19, 20]. In line with their reported advantages, XGBoost and Cubist provided predictions noticeably in agreement with FE results in our study [20]. Algorithms used in previous ML cardiac models could be computationally expensive, and do not provide feature importance [16, 30].

**FIGURE 8 |:**
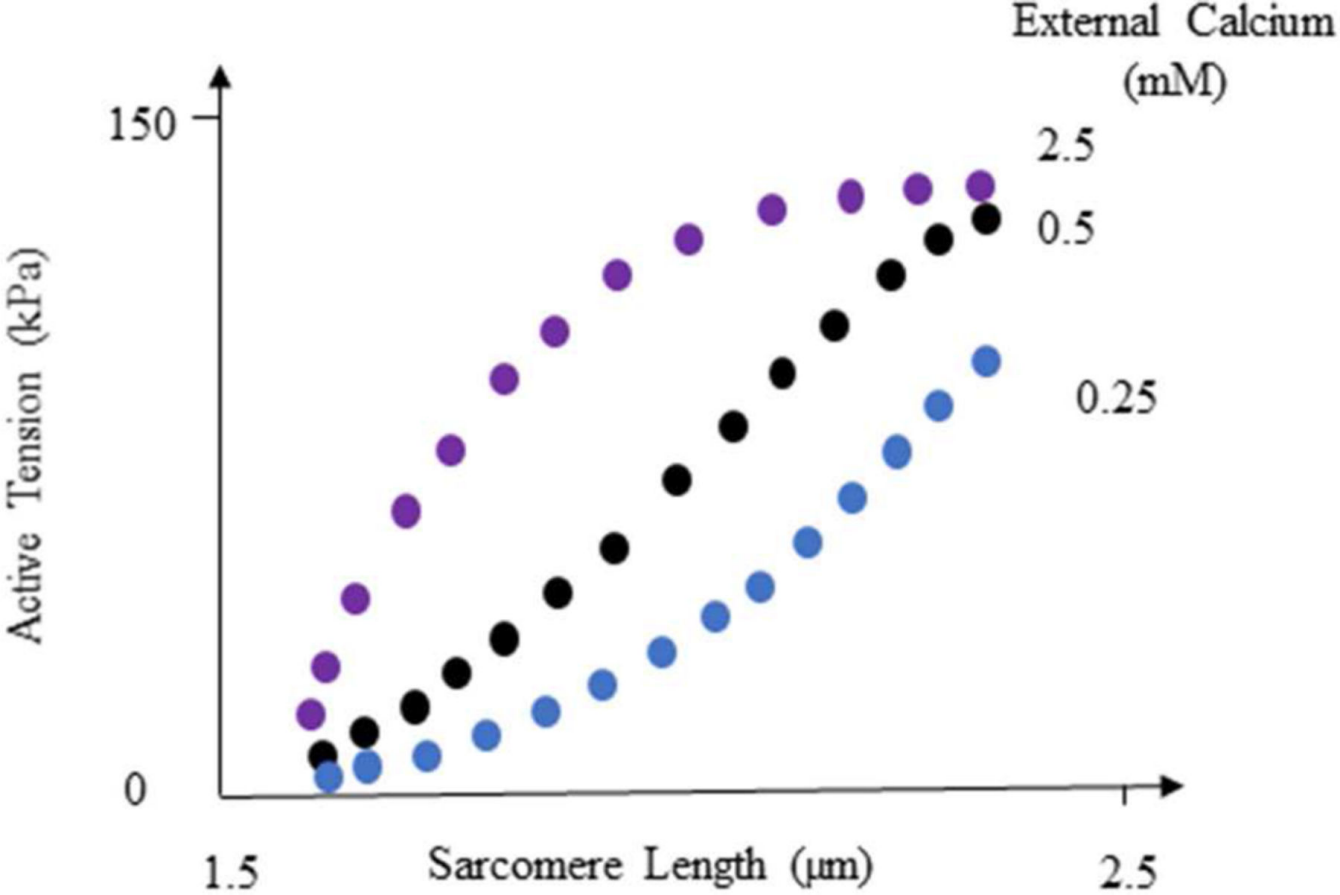
Active tension changes with sarcomere length, reproduced from Guccione and McCulloch [22]. For more information see Guccione and McCulloch [22] and Figure 8 in their paper.

According to ML analysis, the LV pressure and volume are primarily affected by the initial myofiber length (with no active tension) in the right ventricle and left ventricle, respectively (relative to other parameters in Table 3). This result is in line with experimental studies (Figure 8) that reported the active sarcomere tension is related to muscle length [22]. Also, the importance values (Table 3) showed the important role that RV could have in the mechanics of LV. For the passive behavior (Table 3), the coordinates which represent the location of each finite element, have the highest importance in stress predictions. If any of these coordinates alters, the stresses change accordingly (coordinates change with element number).

We used stress data from LV endocardium because endocardium plays an important role in etiology of cardiac diseases. For example, endocardial mechanics is hypothesized to alter over the course of HFpEF development [4, 31–33]. In addition, subendocardial ischemia health conditions reportedly affect LV torsion [34]. However, our ML methodology can be applied to stress in any region of the myocardium.

In our study, we used a set of mechanical properties that were relevant to calibration of active and passive behavior of the myocardium, and all the parameter sets produced converged FE models. Also, the resultant combinations led to physiologically relevant models. For example, after alterations in passive properties, 98.5% of the myofiber stresses were between 0 and 15 kPa, which is in line with published data for LV stress [4, 8, 23]. However, selection of the mechanical properties is not relevant in terms of the applications of ML in prediction of LV mechanics. We could select other parameters in the constitutive equations, but the conclusions of our study would not change.

## Study Limitations and Future Directions

In this ML study, we used data from a single human subject (for pressure and volume) and a single swine model (for stresses), and we did not consider heterogenous properties. The training data used in this study may not capture important aspects of the heart behavior over a broad range of subject-specific or pathological conditions, but our ML model can be trained using data from more subjects and/or pathological conditions. Subject-specific parameters such as anatomy, health conditions, sex, and age change FE models in many respects such as geometry, loads, boundary conditions, and heterogenicity of material parameters. The ML algorithm would have wider applicability if we consider a wider range of subjects, geometries, heterogenous material parameters, and diseased conditions. When the training is performed based on data from different subjects, the mechanical properties obtained from Latin hypercube DOE can also be optimized such that the selected properties better reflect physiological data [16]. These aspects can be included in future developments of our ML methodology.

We showed the applicability of our ML methodology for predicting LV mechanics with examples from LV pressure, volume and stress data. However, the number of test data could be increased to improve the ML model. It is recommended to use 20% of data for testing the ML model [35, 36]. In our study, for stress predictions, the test data were more than 16% of data, and for LV volume and pressure predictions the number of test data was <4% data. When the number of data is limited, an alternative approach is to use cross-validation [35, 36]. Therefore, in our ML models, we implemented cross-validation (Table 1). As a future direction, the number of the data could be increased to include more test examples.

We used the old FE results (Figures 2, 5), despite their limitations: the first “bump” in Figure 2 is too high, and the right lower region in Figure 5 does not correspond to physiological data. These non-physiological FE results were caused by data uncertainty and FE numerical analysis at the time of FE simulations (2015). Material properties, boundary conditions and other aspects of the FE models have likely caused these non-physiological characteristics. We have previously shown the validity of our FE models [4, 7, 8]. In particular, we have developed four-chamber whole heart models that simulate the heart mechanics more realistically than the results shown in Figures 2, 5 [8]. In this paper, we used data shown in Figures 2, 5 because generating new FE results would be time-consuming, and the goal of this paper was better achieved using these old FE results. Specifically, in this paper our focus was on applicability of our ML approach to predict the mechanics of the LV.

There are many aspects of the heart mechanics and behavior that could be fed into our ML algorithm. For example, in patients with HF induced by infraction, the infarcted tissue properties change in different stages of disease/recovery. To estimate the LV wall stress, an ML algorithm can be used wherein LV properties, geometry and pressure are the inputs and wall stresses are the outputs. This strategy can serve as a monitoring tool for optimizing treatment of HF.

Another important aspect of our ML model is its application in LV assistive device design and drug development. Our ML model can give key information such as stresses, strains, pressure and volume in real time. This information (and similar data) are important in the design process of left ventricle assist devices, stents, heart valves, and effects of drugs on LV tissue. Our ML methodology can be used to track the alterations in LV behavior in real time, when the device design iterations or drug safety and efficacy testing are conducted. This approach has been reported in other industries [11].

In this study, we focused on pressure, volume and stresses in the LV. Future studies could include other aspects of the LV or mechanical characteristics of other parts of the heart. For example, the ML model can be trained to predict dP/dt. Also, volume, pressure, stress and strains of the right ventricle can be fed into the ML model. The relevant FE data can be calculated for different healthy, diseased and treated conditions with or without implants and assistive devices. Specifically, we recently developed FE models to calibrate mechanical properties in beating hearts [37]. The LV pressure and volumes can be used as the features of the data sets, and the mechanical properties can be predicted. However, to use our ML methodology for material calibration, more features need to be included, such as sex, age and geometrical or strain data, which were not available in this study. Once the ML is trained for material calibration, the health conditions of normal and diseased hearts can be estimated by implementing our ML approach in portable devices. This line of research is the subject of our future studies.

## CONCLUSIONS

Data such as pressure, volume, and stresses are crucial to understand the health conditions of the heart. Although FE is a powerful tool that can provide this information, it often takes too long for real- time applications. Using ML, these data can be produced in a matter of seconds, and hence ML linked with FE enables using more crucial data in real time. This possibility is important in many areas, including planning surgeries, designing medical devices, and monitoring health conditions of the heart.

## Figures and Tables

**TABLE 1 |:** ML hyperparameters that were altered during grid search analysis.

Parameter	Description*	Values	Best parameters				
			Pressure	Volume	Myofiber stress	Trans-myofiber stress	Shear stress
learning_rate	The learning rate of the boosting	0.01, 0.05, 0.1	0.05	0.05	0.05	0.01	0.1
n_estimators	Number of trees in the model	500, 1,000, 1,500	500	1,500	1,500	1,000	1,500
Max_depth	Maximum depth of trees	7,15,20	15	7	7	7	7

The number of folds in cross validation (cv) = 3.

* XGBoost python package guide.

**TABLE 2 |:** Approximate run time for FE and ML models.

_Model_╲^Output^	Pressure	Volume	Myofiber stress	cross-myofiber stress	Shear stress
ML training (including grid search)	3 CPU hours	12 CPU hours	8 CPU hours	8 CPU hours	7 CPU hours
ML training (using one set of optimized hyper-parameters)	7 CPU minutes	34 CPU minutes	22 CPU minutes	14 CPU minutes	23 CPU minutes
ML test	2 CPU seconds	11 CPU seconds	5 CPU seconds		
FE (a single simulation)	1,000 CPU* hours	1,000 CPU* hours	20 CPU minutes		

* This CPU hour time refers to computations for a four-chamber model in 2015.

**TABLE 3 |:** The ML features and their importance for predicting LV pressure, volume and stresses.

Feature	Feature Description	Importance				
		Pressure	Volume	S_11_	S_22_	S_12_
I_0(RV)_	I_0_ in Equation (6) for RV	0.459	0.188			
t	Time after excitation	0.317	0.225			
I_0(LV)_	I_0_ in Equation (6) for LV	0.113	0.507			
t_0(LV)_	t_0_ in Equation (5) for LV	0.073	0.033			
T_max(LV)_	T_max(LV)_ in Equation (3) for LV	0.018	0.037			
T_max(RV)_	T_max_ in Equation (3) for RV	0.010	0.004			
t_0(RV)_	t_0_ in Equation (5) for RV	0.010	0.005			
theta	Element centroid coordinate in circumferential direction			0.278	0.250	0.434
z	Element centroid coordinate in *z* (longitudinal) direction			0.236	0.222	0.224
r	Element centroid coordinate in radial direction			0.187	0.226	0.180
a	Passive property 1 in Equation (1)			0.087	0.096	0.077
a_s_	Passive property 2 in Equation (1)			0.137	0.049	0.035
a_f_	Passive property 3 in Equation (1)			0.052	0.107	0.029
a_fs_	Passive property 4 in Equation (1)			0.023	0.050	0.021

**TABLE 4 |:** The parameters of LV pressure and volume computed by FE (actual) and ML (predicted).

	Pressure Average ± SD			Volume Average ± SD		
	FE	ML	%Error	FE	ML	%Error
Minimum	20.4 ± 4.5 mmHg	19.1 ± 4.0 mmHg	5.6 ± 5.1	106.5 ± 13.4 ml	107.1 ± 13.1 ml	0.6 ± 1.9
Maximum	61.3 ± 20.8 mmHg	63.2 ± 20.5 mmHg	3.6 ± 1.6	139.9 ± 5.8 ml	139.7 ± 5.3ml	0.1 ± 0.46
Time to maximum	0.3 ± 0.04s	0.3 ± 0.03s	0.9 ± 1.3s	0.2 ± 0.0s	0.2 ± 0.01 s	4.6 ± 7.6
Maximum time derivative	792.7 ± 518.8 mmHg/s	1848.6 ± 664.6 mmHg/s	184.8 ± 84.3	221.1 ± 80.1 ml/s	664.9 ± 423.2 ml/s	211.7 ± 155.1

These numbers show the average and standard deviation (SD) for three cycles in Figure 2 (pressure) and Figure 4 (volume).

**TABLE 5 |:** *R*^2^ and MAE Score for the ML predictions.

Model	*R*^2^ score (average ± SD)	MAE (average ± SD)
Pressure (Cubist)	0.958 ± 0.029	1.623 ± 0.191 mmHg
Pressure (XGBoost)	0.939 ± 0.067	1.544 ± 0.298 mmHg
Volume (Cubist)	0.942 ± 0.055	1.495 ± 0.260 ml
Volume (XGBoost)	0.923 ± 0.050	1.734 ± 0.584 ml
Myofiber stress (XGBoost)	0.971 ± 0.040	0.334 ± 0.228 kPa
Trans-myofiber stress (XGBoost)	0.936 ± 0.042	0.075 ± 0.024 kPa
Shear stress (XGBoost)	0.994 ± 0.006	0.050 ± 0.032 kPa

For each single cardiac cycle (pressure and volume) or LV (stress) test sample the error was calculated and then, the average and standard deviation was calculated for all test samples.
